# The genetic relationship between immune competence traits and micro-genetic environmental sensitivity of weight, fat, and muscle traits in Australian Angus cattle

**DOI:** 10.1186/s12711-025-00998-8

**Published:** 2025-09-25

**Authors:** Mette D. Madsen, Julius H. J. van der Werf, Aaron Ingham, Brad Hine, Antonio Reverter, Sam A. Clark

**Affiliations:** 1 School of Environmental and Rural Science, University of New England, Armidale, NSW 2351 Australia; 2 Queensland Biosecurity Precinct, Agriculture & Food, CSIRO, St Lucia, QLD 4067 Australia; 3 F.D. McMaster Laboratory, Agriculture & Food, CSIRO, Armidale, NSW 2350 Australia

## Abstract

**Background:**

Improving immune competence (IC) in livestock could reduce the incidence of disease and reliance on the use of antibiotics. In Australian Angus cattle, IC is a measure of an animal’s combined ability to mount antibody and cell-mediated immune responses (AMIR and CMIR). Immune competence may affect traits such as growth and related phenotypes as well as the variability of such phenotypes. However, the genetic relationship between IC and genetic sensitivity to individual environments, measured as micro-genetic environmental sensitivity (GES), is yet to be reported. In this study the genetic parameters of, and correlations between, AMIR or CMIR and micro-GES of live weaning weight (WW) and ultrasound scan records of rib (RIB) and rump (RUMP) fat depth and eye muscle area (EMA) measured between 501 and 900 days of age were estimated. This was accomplished by fitting eight multivariate models with AMIR or CMIR and a double hierarchical generalised linear model on a production trait.

**Results:**

The heritabilities were 0.35 and 0.36 for AMIR and CMIR, respectively, and 0.25–0.70 for the production traits. The heritabilities and the genetic coefficient of variation of micro-GES of the production traits ranged from 0.01–0.04 and 18–82%, respectively, and were higher in RIB and RUMP than WW and EMA. The genetic correlations between AMIR and WW, RIB, RUMP, or EMA were -0.35 (SE 0.11), 0.11 (0.12), 0.06 (0.12) and -0.13 (0.12), respectively, while the genetic correlations between CMIR and WW, RIB, RUMP, or EMA were -0.26 (0.12), 0.15 (0.13), 0.16 (0.12) and 0.04 (0.13), respectively. The genetic correlations between IC and micro-GES of WW, RIB, RUMP or EMA were moderately negative to lowly positive and had large SEs rendering them non-significant.

**Conclusions:**

The unfavourable genetic correlation between the IC traits and WW supports the hypothesis that mounting an effective immune response can direct resources away from growth when resources are limited. Based on the heritabilities and genetic coefficient of variation of micro-GES, selection to increase uniformity is possible for WW, RIB, RUMP and EMA. The standard errors of the genetic correlations between IC and micro-GES of the production traits were too large to draw any definite conclusions about their relationships. Standard errors are expected to reduce as more IC records are collected.

**Supplementary Information:**

The online version contains supplementary material available at 10.1186/s12711-025-00998-8.

## Background

Disease outbreaks have major health, welfare, and performance consequences for livestock. In many production systems, animals will be exposed to a multitude of pathogens that may cause infections. Some infections require interventions for animals to recover, while others are eliminated by the animals’ immune system alone. Improving immune competence of livestock is expected to increase the ability of animals to cope with disease challenges leading to fewer serious disease outbreaks, reduced health-related costs and improve the health and welfare of animals [1]. As an additional benefit of improving animal health, reliance on antibiotics to treat disease will be reduced, improving consumer trust in products from livestock [1]. The increase in consumer trust will stem from alleviating the consumer concerns about the use of antibiotics in production animals, which has been perceived to contribute to the global issue of antimicrobial resistance [2]. In livestock production systems, the inclusion of immune competence (IC) traits in the breeding goal is therefore desirable.

In Australian Angus, methods for measuring IC traits in the form of antibody and cell-mediated immune response (AMIR and CMIR, respectively) have been developed [1]. The heritabilities of AMIR and CMIR in Australian Angus have been reported in the range of 0.25–0.42 [1, 3–5]. The heritability of IC indexes based on AMIR and CMIR have been reported in the range of 0.28–0.42 [4, 5]. These results indicated that selection to alter IC in the population is possible.

Selection to improve IC is likely to cause correlated responses in other traits. It has been shown that intense selection for growth negatively impacted immune function in selection lines of broiler chickens [6]. Similarly, immune competence and weaning weight have been shown to have weak unfavourable genetic correlations in Australian Angus cattle [5]. It is possible that the unfavourable correlation in IC when selecting for increased growth is due to resource allocation. Mounting immune responses requires resources, such as energy, regulatory cytokines and chemokines, generation of immune cells, etc. If resources are limited, these resources may be diverted from other processes, affecting traits of key interest in the breeding program [6–8]. It would therefore be reasonable to expect unfavourable genetic correlations between immune competence and not only weight traits but also carcase traits such as fat depth, muscle size etc.

Estimation of correlation between traits generally focuses on the correlation between mean performances. However, correlations may also exist between the individual mean performance of one trait, such as IC, and the individual variability of another, such as growth. It is possible that IC may be related to the variability among phenotypes of other traits due to differences in resource allocation among animals. For some traits, individual variability of phenotypes can be heritable, in which case the trait exhibits micro-genetic environmental sensitivity (GES). Micro-GES is a form of genotype by environment interaction in response to the animal’s individual environments, termed micro-environments [9]. Because micro-environments are individual and transient, they are unmeasurable, and little is known about what causes micro-GES. External fluctuations in biotic and abiotic factors, as well as internal changes in subcellular components, are thought to contribute to micro-GES [10]. Differences in resource allocation between growth and IC could possibly be a cause of micro-GES. There may be genetic variation in how animals experiencing limited resources divert resources away from a specific trait or a set of traits. Genotypes that are better able to limit this resource loss may be more robust to variation in micro-environments, therefore showing less variability in phenotype.

The aim of this study was to estimate the genetic variance due to micro-GES of live weaning weight (WW) and ultrasound scan records of rib (RIB) and rump (RUMP) fat depth and eye muscle area (EMA) in Australian Angus cattle, and the genetic relationship between micro-GES of these production traits and the IC traits.

## Methods

### Immune response trait definitions

Immune competence phenotypes for individual animals were provided by CSIRO and Angus Australia. Immune competence phenotypes were assessed using the protocol described by Hine et al. [1]. In brief, calves were subcutaneously vaccinated (high on the neck) with a 7in1 clostridial and leptospira multivalent vaccine (Ultravac 7in1, Zoetis) as per manufacturer’s recommendations at weaning. Production of antigen-specific serum IgG1 antibody in response to vaccination was used as a measure of the animal’s ability to mount AMIR. Measures of AMIR represent square root transformed optical density values generated using an enzyme-linked immunosorbent assay (ELISA) corrected for inter-plate variation. The magnitude of delayed-type hypersensitivity reactions to vaccine injected intradermally into the caudal fold of the tail was used as a measure of the animal’s ability to mount CMIR. Measures of CMIR represent the log-transformed ratio between the double skinfold thickness at test and control site (intradermal saline injection) at 48 h post-injection. The log-transformed ratio between the double skinfold thickness at test and control site measured prior to injection was used in analysis as a covariate to account for initial double skinfold thickness.

### Weight, fat, and muscle trait definitions

Angus Australia provided records of weaning weight (WW, also known as 200-day weight) measured in kg on live animals between 81 and 300 days of age. Also provided were records of eye muscle area (EMA), rib fat (RIB) and rump fat (RUMP) measured via ultrasound scanning of live animals between 501 and 900 days of age. EMA and RIB were measured on the longissimus dorsi muscle between 12 and 13th rib and represent the cross-sectional area in cm^2^ and fat depth in mm, respectively, while RUMP was the fat depth in mm at the intersection between the lines drawn from the high bone and the pin bone [11].

### Data editing

Quality control was carried out such that the records included in the final dataset all had known sire, dam, birth date, birth year, sex, herd, date of observation, mating type (natural or artificial insemination), embryo transfer status (true or false), recipient dam for embryo transfer calves, age of dam (dam or recipient dam). Furthermore, records were only kept if the animal was born in 2012 or later, rearing dams were between 1.5 and 15 years of age, birth type (single or twins) were the same as the number of calves reared by the same (recipient) dam, and the phenotype was within 3 phenotypic SDs from their contemporary group (CG) mean.

For the IC records, CG were constructed by concatenating herd, year, and test cohort. CGs were the same for AMIR and CMIR. For WW, RIB, RUMP and EMA, trait specific CGs were constructed by concatenating herd, birth year, observation date for the trait in question, breeder defined management group, birth type and embryo transfer status. Age slicing further subdivided CGs for WW, RIB, RUMP, and EMA. Age slices covered 45 days for WW and 60 days for RIB, RUMP, and EMA as per Graser et al. [12], and slices were symmetric around the average age of the CG. Age slicing was not used in CGs for the IC traits. An iterative filtration was done for each trait to ensure that sires had at least 5 offspring across at least 5 CGs and that CGs contained at least 5 animals. All animals with a weaning weight record retained in the dataset for analysis also had either an ultrasound scan performance record or IC phenotype. Summary statistics for each trait can be found in Table 1.

**Table 1:** Summary statistics for the analysed dataset

Parameter	Statistic	WW	RIB	RUMP	EMA	AMIR	CMIR
Records	Count	31,699	83,034	83,314	83,486	3910	3908
Phenotype	Mean (SD)	254.60 (51.63)	6.11 (2.76)	7.89 (3.73)	80.49 (17.56)	0.89 (0.43)	0.61 (0.42)
	Range	77–496	1–22	1–33	31–157	0.01–2.13	0.85–4.92
Sire	Count	1378	2985	2988	2988	264	264
	Mean (SD)	23.00 (38.86)	27.82 (70.42)	27.88 (70.53)	27.94 (70.76)	14.81 (7.79)	14.80 (7.80)
	Range	5–477	5–1314	5–1315	5–1317	5–55	5–55
Rearing dam	Count	23,138	53,748	53,904	53,964	2872	2870
	Mean (SD)	1.37 (0.74)	1.54 (0.93)	1.55 (0.93)	1.55 (0.93)	1.36 (0.66)	1.36 (0.66)
	Range	1–7	1–9	1–9	1–9	1–5	1–5

WW, weaning weight (kg); RIB,  rib fat (mm); RUMP, rump fat (mm); EMA, eye muscle area (cm^2^); AMIR, antibody-mediated immune response $\left( {\sqrt {optical\;density} } \right)$; CMIR, cell-mediated immune response (log ratio test/control reaction 48 h post injection (mm))

### Pedigree

For analysis, two pedigrees were constructed based on the sires (sire pedigree) and on the rearing dam (dam pedigree) of animals with records. The pedigrees were limited to animals born from 1990 onwards and traced 3 generations back from sires or dams of animals with records using DmuTrace v2 [13]. The sire pedigree contained 10,948 animals and the dam pedigree contained 98,151 animals.

### Statistical analysis

To estimate the variance components of AMIR, CMIR, WW, RIB, RUMP and EMA as well as micro-GES of WW, RIB, RUMP and EMA, a multivariate model was fitted with a double hierarchical generalised linear model (DHGLM) on the production trait for each combination of IC and production trait.

A DHGLM consists of two interdependent parts, a mean part and a dispersion part, fitted as a bivariate model for a single measured trait [14]. Thus, models for IC and micro-GES of the production traits were trivariate models. Each of these trivariate models allowed for simultaneous estimation of the variance components of the IC trait and the mean and dispersion of the production trait as well as the covariance between these components.

The trivariate models were fitted as sire models, treating the records of a sire’s offspring as repeated records of the sire. This was done because WW, RIB, RUMP, and EMA were all single recorded traits and DHGLMs for single recorded traits fitted as animal models have been shown to result in biased estimates [15].

The fixed effects and covariates for each trait were chosen using the ‘lmer’ function of the lme4 package [16] in R [17]. The tested and included fixed effects and covariates are listed in Table 2. It is common to treat RIB, RUMP and EMA as different traits in bulls and heifers/steers, however due to the complexity of the DHGLMs, conversion issues occurred when attempting to treat the scan traits as different in bulls and heifers/steers. Sex was therefore included as a fixed effect in the models. For WW, RIB, RUMP and EMA, the same fixed effects and covariates were included in the mean and dispersion. Maternal genetic and permanent environment effects were included in the model for WW but not for RIB, RUMP or EMA as recommended by Graser et al. [12]. For AMIR and CMIR, maternal genetic and permanent environmental effects were tested but the inclusion of these effects did not improve model fit relative to the models excluding the maternal effects based on the Akaike Information Criterion. Consequently, the most parsimonious models were chosen.

**Table 2:** Tested and included fixed effects and covariates

Trait	Fixed effects		Covariates				
	Sex	CG	Age	Age^2^	DA	DA^2^	T0
WW	✓	✓	✓	–	✓	✓	nt
RIB	✓	✓	✓	–	–	–	nt
RUMP	✓	✓	✓	–	–	–	nt
EMA	✓	✓	✓	–	–	–	nt
AMIR	✓	✓	–	–	–	–	nt
CMIR	–	✓	–	–	–	–	✓

WW, weaning weight; RIB, rib fat; RUMP, rump fat; EMA, eye muscle area; AMIR, antibody-mediated immune response; CMIR, cell-mediated immune response; CG, contemporary group; Age, age at measurement; Age^2^, squared age at measurement; DA, dam age at calving; DA^2^, squared dam age at calving; T0, log (double skinfold thickness at test site/double skinfold thickness at control site); ✓, included in model; –, not included; nt, not tested

To obtain variance components of, and genetic correlations between, either AMIR or CMIR and both mean and dispersion of either WW, RIB, RUMP, or EMA, 8 trivariate models were run. The general model was:

$$\left[ {\begin{array}{*{20}c} {{\mathbf{y}}_{{{\mathbf{IC}}}} } \\ {{\mathbf{y}}_{{\mathbf{m}}} } \\ {{\mathbf{y}}_{{\mathbf{d}}} } \\ \end{array} } \right] = \left[ {\begin{array}{*{20}c} {{\mathbf{X}}_{{{\mathbf{IC}}}} } & {\mathbf{0}} & {\mathbf{0}} \\ {\mathbf{0}} & {{\mathbf{X}}_{{\mathbf{m}}} } & {\mathbf{0}} \\ {\mathbf{0}} & {\mathbf{0}} & {{\mathbf{X}}_{{\mathbf{d}}} } \\ \end{array} } \right]\left[ {\begin{array}{*{20}c} {{\mathbf{b}}_{{{\mathbf{IC}}}} } \\ {{\mathbf{b}}_{{\mathbf{m}}} } \\ {{\mathbf{b}}_{{\mathbf{d}}} } \\ \end{array} } \right] + \left[ {\begin{array}{*{20}c} {{\mathbf{Z}}_{{{\mathbf{IC}}}} } & {\mathbf{0}} & {\mathbf{0}} \\ {\mathbf{0}} & {{\mathbf{Z}}_{{\mathbf{m}}} } & {\mathbf{0}} \\ {\mathbf{0}} & {\mathbf{0}} & {{\mathbf{Z}}_{{\mathbf{d}}} } \\ \end{array} } \right]\left[ {\begin{array}{*{20}c} {{\mathbf{s}}_{{{\mathbf{IC}}}} } \\ {{\mathbf{s}}_{{\mathbf{m}}} } \\ {{\mathbf{s}}_{{\mathbf{d}}} } \\ \end{array} } \right] + \left[ {\begin{array}{*{20}c} {{\mathbf{e}}_{{{\mathbf{IC}}}} } \\ {{\mathbf{e}}_{{\mathbf{m}}} } \\ {{\mathbf{e}}_{{\mathbf{d}}} } \\ \end{array} } \right]$$

where ${\mathbf{y}}_{\mathbf{I}\mathbf{C}}$ contained the AMIR or CMIR phenotypes, ${\mathbf{y}}_{\mathbf{m}}$ contained the measured phenotypes for the mean part of the DHGLM on WW, RIB, RUMP, or EMA, and ${\mathbf{y}}_{\mathbf{d}}$ was the calculated phenotype (see Eq. (5)) for the dispersion part of the DHGLM on WW, RIB, RUMP, or EMA. ${\mathbf{b}}_{\mathbf{I}\mathbf{C}}$, ${\mathbf{b}}_{\mathbf{m}}$ and ${\mathbf{b}}_{\mathbf{d}}$ contained the fixed effects listed in Table 2 for the IC trait and the mean and dispersion part of the DHGLM, respectively. ${\mathbf{X}}_{\mathbf{I}\mathbf{C}}$, ${\mathbf{X}}_{\mathbf{m}}$ and ${\mathbf{X}}_{\mathbf{d}}$ were the incident matrices relating phenotypes to ${\mathbf{b}}_{\mathbf{I}\mathbf{C}}$, ${\mathbf{b}}_{\mathbf{m}}$ and ${\mathbf{b}}_{\mathbf{d}}$, respectively. ${\mathbf{s}}_{\mathbf{I}\mathbf{C}}$ was the random genetic sire effect for the IC trait, and ${\mathbf{s}}_{\mathbf{m}}$ and ${\mathbf{s}}_{\mathbf{d}}$ was the random genetic sire effect in the mean and dispersion part of the DHGLM, respectively. ${\mathbf{Z}}_{\mathbf{I}\mathbf{C}}$, ${\mathbf{Z}}_{\mathbf{m}}$ and ${\mathbf{Z}}_{\mathbf{d}}$ were the incidence matrices relating phenotypes to ${\mathbf{s}}_{\mathbf{I}\mathbf{C}}$, ${\mathbf{s}}_{\mathbf{m}}$ and ${\mathbf{s}}_{\mathbf{d}}$, respectively. The distribution assumptions for the random genetic sire effects were $$\left[ {\begin{array}{*{20}c} {{\mathbf{s}}_{{{\mathbf{IC}}}} } \\ {{\mathbf{s}}_{{\mathbf{m}}} } \\ {{\mathbf{s}}_{{\mathbf{d}}} } \\ \end{array} } \right]\sim MVN\left( {\left[ {\begin{array}{*{20}c} {\mathbf{0}} \\ {\mathbf{0}} \\ {\mathbf{0}} \\ \end{array} } \right],\left[ {\begin{array}{*{20}c} {\sigma_{{s_{IC} }}^{2} } & {\sigma_{{s_{IC} s_{m} }} } & {\sigma_{{s_{IC} s_{d} }} } \\ {\sigma_{{s_{m} s_{IC} }} } & {\sigma_{{s_{m} }}^{2} } & {\sigma_{{s_{m} s_{d} }} } \\ {\sigma_{{s_{d} s_{IC} }} } & {\sigma_{{s_{d} s_{m} }} } & {\sigma_{{s_{d} }}^{2} } \\ \end{array} } \right] \otimes {\mathbf{A}}} \right)$$, where $\mathbf{A}$ was the relationship matrix based on the sire pedigree and $\otimes$ was the Kronecker product. ${\mathbf{e}}_{\mathbf{I}\mathbf{C}}$, ${\mathbf{e}}_{\mathbf{m}}$ and ${\mathbf{e}}_{\mathbf{d}}$ were the residuals of IC, the mean and dispersion part of the DHGLM on the production trait, respectively, with distribution assumptions of $$\left[ {\begin{array}{*{20}c} {{\mathbf{e}}_{{{\mathbf{IC}}}} } \\ {{\mathbf{e}}_{{\mathbf{m}}} } \\ {{\mathbf{e}}_{{\mathbf{d}}} } \\ \end{array} } \right]\sim MVN\left( {\left[ {\begin{array}{*{20}c} 0 \\ 0 \\ 0 \\ \end{array} } \right],\left[ {\begin{array}{*{20}c} {{\mathbf{I}}\sigma_{{e_{IC} }}^{2} } & 0 & 0 \\ 0 & {{\mathbf{W}}_{{\mathbf{m}}}^{ - 1} \sigma_{{ \in_{m} }}^{2} } & 0 \\ 0 & 0 & {{\mathbf{W}}_{{\mathbf{d}}}^{ - 1} \sigma_{{ \in_{d} }}^{2} } \\ \end{array} } \right]} \right)$$, where $\mathbf{I}$ was an identity matrix of appropriate size, and ${\mathbf{W}}_{\mathbf{m}}$ and ${\mathbf{W}}_{\mathbf{d}}$ were matrices containing weights for the residual variances of the mean and dispersion part of the DHGLM, respectively (see Eq. (1) and (2)).

For WW, the model was extended to include random maternal genetic ($\mathbf{c}$) and permanent environment ($\mathbf{p}\mathbf{e}$) effects on the mean part of the DHGLM. The distribution assumptions for $\mathbf{c}$ and $\mathbf{p}\mathbf{e}$ were ${\mathbf{c}}\sim MVN\left( {{\mathbf{0}},\sigma_{c}^{2} \otimes {\mathbf{A}}_{{\mathbf{D}}} } \right)$ and $\mathbf{p}\mathbf{e}\sim N\left(0,\mathbf{I}{\sigma }_{pe}^{2}\right)$, respectively, where ${\mathbf{A}}_{\mathbf{D}}$ was the relationship matrix based on the dam pedigree and $\mathbf{I}$ was an identity matrix of appropriate size.

The calculations of dispersion phenotypes and weights for the residuals in the DHGLMs were the same for WW, RIB, RUMP, and EMA.

The weight for the residual variances for ${\mathbf{y}}_{\mathbf{m}}$ and ${\mathbf{y}}_{\mathbf{d}}$ were:

1 $${\mathbf{W}} = diag\left( {\widehat{{{\mathbf{y}}_{{\mathbf{d}}} }}} \right)^{ - 1}$$

2 $${\mathbf{W}}_{{\mathbf{d}}} = \frac{{1 - {\mathbf{h}}}}{2}$$

where $\mathbf{h}$ was a vector of the diagonal elements of the hat-matrix of ${\mathbf{y}}_{\mathbf{m}}$ ($\widehat{{\mathbf{y}}_{\mathbf{m}}}=\mathbf{H}{\mathbf{y}}_{\mathbf{m}}$) also known as the leverage [18]. ${\sigma }_{{\in }_{m}}^{2}$ and ${\sigma }_{{\in }_{d}}^{2}$ were expected to equal 1, because the weights contained the reciprocal of the residual variance per observation. However, estimating them allows for more flexibility in the model [15]. The residual variances (${\sigma }_{{e}_{m}}^{2}$ and ${\sigma }_{{e}_{d}}^{2}$) were then

3 $$\sigma_{{e_{m} }}^{2} = \frac{{\sigma_{{ \in_{m} }}^{2} }}{{\frac{{tr\left( {{\mathbf{W}}_{{\mathbf{m}}} } \right)}}{n}}}$$

4 $$\sigma_{{e_{d} }}^{2} = \frac{{\sigma_{{ \in_{d} }}^{2} }}{{\frac{{tr\left( {{\mathbf{W}}_{{\mathbf{d}}} } \right)}}{n}}}$$

where $n$ is the total number of records for the trait in question.

The phenotypes used to estimate micro-GES (${\mathbf{y}}_{\mathbf{d}}$) were the squared estimated residuals of ${\mathbf{y}}_{\mathbf{m}}$ calculated via

5 $$y_{di} = \frac{{\widehat{{e_{m} }}_{i}^{2} }}{{1 - h_{i} }}$$

where ${\widehat{e}}_{i}^{2}$ was the squared estimated residual of observation *i* and ${h}_{i}$ was the leverage of observation *i* [19].

All models were analysed using DMU [20] using the following algorithm.

1. Initialize model by running a bivariate sire model on either AMIR or CMIR and either WW, RIB, RUMP, or EMA with homogeneous residual variance.
2. Calculate ${\mathbf{y}}_{\mathbf{d}}$ from Eq. (5) and ${\mathbf{W}}_{\mathbf{d}}$ from Eq. (2) based on ${\widehat{\mathbf{e}}}_{\rm m}$ and $\mathbf{h}$ estimated in step 1.
3. Run a univariate generalized linear mixed model on ${\mathbf{y}}_{\mathbf{d}}$ with a log-link function.
4. Calculate ${\mathbf{W}}_{\mathbf{m}}$ from Eq. (1) based on $\widehat{{\mathbf{y}}_{\mathbf{d}}}$ estimated in step 3.
5. Run the trivariate model
6. Update ${\mathbf{W}}_{\mathbf{m}}$, ${\mathbf{y}}_{\mathbf{d}}$, and ${\mathbf{W}}_{\mathbf{d}}$ based on $\widehat{{\mathbf{y}}_{\mathbf{d}}}$, $\widehat{{\mathbf{e}}_{\mathbf{m}}}$ and $\mathbf{h}$ estimated in step 5.
7. Iterate steps 5–6 until converged or the algorithm has run 50 iterations.

Convergence was said to have been reached when the maximum absolute difference in estimates between the current and previous run were less than 10^–3^. If the algorithm ran 50 iterations, the model was considered as not converged and results were discarded. See Additional file 1 for a guide for how to run the above algorithm using R and DMU.

### Post-analysis corrections and heritabilities

As the models were implemented as sire models, the additive genetic sire variance (${\sigma }_{s}^{2}$) was estimated rather than the additive genetic variance (${\sigma }_{a}^{2}$). The additive genetic sire variance is only ¼${\sigma }_{a}^{2}$ and the remaining ¾${\sigma }_{a}^{2}$ is included in the estimated residual variance. For AMIR, CMIR and the mean part of the DHGLM fitted to WW, RIB, RUMP, and EMA the additive genetic variance was 4 times the sire variance. The heritability (${h}^{2}$) was the ratio of additive genetic variance to phenotypic variance (${\sigma }_{P}^{2}$, sum of the variances of all random effects).

For the dispersion part of WW, RIB, RUMP and EMA, the corrections to obtain the additive genetic variance had to consider the fact that ¾ of the additive genetic variance of the mean part was included in the residual variance of the mean part used to calculate the dispersion phenotype. To correct for the additional genetic component in the dispersion, the additive genetic variance of the dispersion was calculated as described in Madsen et al. [31]

6 $$\sigma_{{a_{d} }}^{2} = 4\sigma_{{s_{d} }}^{2} \left( {\frac{{\sigma_{{e_{m} }}^{2} }}{{\sigma_{{e_{m} }}^{2} - 3*\sigma_{{s_{m} }}^{2} }}} \right)^{2}$$

The heritability of the dispersion on the log-scale (${h}_{d}^{2}$) was then the ratio of the additive genetic variance of the dispersion to the phenotypic variance of the dispersion.

The estimated variances of the mean and dispersion were on different scales because the dispersion was fitted with a log-link function. The estimated additive genetic variance of the dispersion can be converted from the log-scale to the scale of measurement [21]. However, the dispersion part of DHGLMs estimates the genetic variance of the residual variance from the mean part of the models. This means the additive genetic variance on the measurement scale has units equal to the measurement unit to the power of 4, e.g., for WW the measurement unit is kg, resulting in the mean part having variance components with kg^2^ as units and the unit of the additive genetic variance of the dispersion on the measurement scale was therefore kg^4^. To make comparison between the mean and dispersion part of DHGLMs more straightforward, the additive genetic SD on the measurement scale was used in the current study:

7 $$\sigma_{{a_{d} }}^{*} = \sqrt {\sigma_{{a_{d} }}^{{2^{*} }} } = \sqrt {2\left( {\sigma_{{e_{m} }}^{2} - 3\sigma_{{s_{m} }}^{2} } \right)^{2} h_{d}^{2} }$$

The heritability on the scale of measurement (${h}_{d}^{{2}^{*}}$) was calculated following Mulder et al. [22]

8 $$h_{d}^{{2^{*} }} = \frac{{\sigma_{{a_{d} }}^{{2^{*} }} }}{{2\sigma_{{P_{m} }}^{4} + 3\sigma_{{a_{d} }}^{{2^{*} }} }}$$

where ${\sigma }_{{P}_{m}}^{4}$ was the squared phenotypic variance of the mean part of the DHGLM.

To estimate the evolvability of the IC and production traits the genetic coefficient of variation for the dispersion of the DHGLMs was calculated following Houle [23].

9 $$GCV = \frac{{\sigma_{a} }}{{\overline{y}}}$$

where ${\sigma }_{a}$ was the genetic (SD) of the trait and $\overline{y}$ was the phenotypic mean of the trait. The evolvability of a trait is a measure of the potential for changing the population mean through selection, while the heritability is a measure of how fast change can occur [23].

For micro-GES the genetic coefficient of variation for the dispersion of the DHGLMs was calculated following Mulder et al. [21]

10 $$GCV_{d} = \frac{{\sigma_{{a_{d} }}^{*} }}{{\sigma_{{e_{m} }}^{2} }}$$

The calculations were conducted using the deltaMethod function of the car package [24], in R [17], which also provides approximate standard errors of all calculations.

### Model consistency

The consistency of models was assessed by comparing estimated variances, heritabilities and genetic correlations between the trivariate model including a DHGLM, and bivariate models for either WW, RIB, RUMP, or EMA along with either AMIR or CMIR. These models were fitted as both sire and animal models and included the same fixed effects as listed in Table 2.

By comparing bivariate sire and animal models, it was possible to determine if any inconsistencies in estimated or calculated parameters were due to fitting a sire model rather than an animal model or due to including a DHGLM on the production trait.

## Results

In this section, only the results from the trivariate models with a DHGLM are presented. The results from the bivariate sire and animal models on WW, RIB, RUMP, or EMA and either AMIR or CMIR can be found in Additional file 2 Tables S1, 2.

### Variances and heritabilities

Estimated additive genetic, residual, maternal genetic and permanent environmental variances and heritabilities of AMIR, CMIR, WW, RIB, RUMP, and EMA and of the dispersion of WW, RIB, RUMP, and EMA were consistent across models, with SD across models less than 4% of the average value. Results were therefore presented as averages. Raw estimates (and their SEs) from each model can be found in Additional file 2 Table S3.

The estimated additive genetic variance of the mean and dispersion was significant (based on 95% confidence intervals of raw estimates) for all production traits, as were the estimated additive genetic variances of AMIR and CMIR (Table 3). Relative to the additive genetic variance of the mean, the additive genetic variance of the dispersion on the log-scale was 1% for WW, 65% for RIB, 37% for RUMP, and 0% for EMA, while the genetic SD of the dispersion on the measurement scale was 35, 85, 90 and 52% of the mean for WW, RIB, RUMP, and EMA, respectively.

**Table 3:** Estimated additive genetic variances and heritabilities averaged across models

	AMIR	CMIR	WW	RIB	RUMP	EMA
${\sigma }_{a}^{2}$	0.02 (0.00)	0.01 (0.00)	128.00 (10.90)	0.32 (0.02)	0.70 (0.04)	6.35 (0.40)
${\sigma }_{{a}_{d}}^{2}$			1.14 (0.41)	0.21 (0.03)	0.26 (0.03)	0.03 (0.01)
${\sigma }_{{a}_{d}}^{*}$			44.66 (3.67)	0.27 (0.01)	0.63 (0.03)	3.32 (0.53)
${\sigma }_{c}^{2}$			57.57 (5.44)			
${\sigma }_{pe}^{2}$			61.02 (5.28)			
${\sigma }_{e}^{2}$	0.03 (0.00)	0.02 (0.00)	54.57 (8.55)	0.61 (0.02)	1.30 (0.03)	18.91 (0.33)
${\sigma }_{{e}_{d}}^{2}$			2.15 (0.03)	1.79 (0.01)	1.80 (0.01)	1.98 (0.01)
${h}^{2}$	0.36 (0.06)	0.36 (0.06)	0.70 (0.05)	0.35 (0.02)	0.35 (0.02)	0.25 (0.02)
${h}_{d}^{2}$			0.45 (0.14)	0.11 (0.01)	0.13 (0.02)	0.02 (0.01)
${h}_{d}^{{2}^{*}}$			0.03 (0.00)	0.04 (0.00)	0.04 (0.00)	0.01 (0.00)
$GCV$	0.14 (0.01)	0.18 (0.01)	0.04 (0.00)	0.09 (0.00)	0.11 (0.00)	0.03 (0.00)
${GCV}_{d}$			0.82 (0.10)	0.45 (0.03)	0.49 (0.03)	0.18 (0.03)

WW, weaning weight; RIB, rib fat; RUMP, rump fat; EMA, eye muscle area; AMIR, antibody-mediated immune response; CMIR, cell-mediated immune response. ${\sigma }_{a}^{2}$, ${\sigma }_{e}^{2}$, ${h}^{2}$ and $GCV$ = additive genetic variance on the animal level, residual variance, heritability and genetic coefficient of variation, respectively, of AMIR, CMIR and the mean of WW, RIB, RUMP, and EMA. ${\sigma }_{{a}_{d}}^{2}$, ${\sigma }_{{e}_{d}}^{2}$ and ${h}_{d}^{2}$ = additive genetic variance on the animal level, residual variance, and heritability, respectively, of the dispersion of WW, RIB, RUMP and EMA on the log-scale. ${\sigma }_{{a}_{d}}^{*}$, ${h}_{d}^{{2}^{*}}$ and ${GCV}_{d}$ = additive genetic SD on the animal level, heritability and genetic coefficient of variation of the dispersion of WW, RIB, RUMP, and EMA on the measurement scale. ${\sigma }_{c}^{2}$ and ${\sigma }_{pe}^{2}$ = the maternal genetic and permanent environmental variance, respectively, of the mean of WW

The dispersion was lowly-moderately heritable for WW, RIB, RUMP, and EMA on the log-scale and lowly heritable on the measurement scale, while the heritability of AMIR, CMIR and the mean of WW, RIB, RUMP, and EMA were all moderate.

The genetic coefficient of variation was low for the IC and production traits. The genetic coefficient of variation of the dispersion of the production traits was moderate-high, and consistently higher than the genetic coefficient of variation of the production trait itself.

In the models with WW, the percentage of phenotypic variance explained by the maternal genetic or permanent environment effect was 19% and 20%, respectively.

### Genetic correlations

The genetic correlation between the mean of the DHGLM and AMIR or CMIR was moderately negative for WW (Table 4). The genetic correlation between the mean and AMIR, or CMIR, was weakly positive for both RIB and RUMP. For EMA, the genetic correlation between the mean and AMIR was weakly negative while the genetic correlation was weakly positive between the mean of EMA and CMIR. The genetic correlation between AMIR or CMIR and the mean of the DHGLM were significantly different from 0 for WW. Genetic correlations between CMIR and the mean of the DHGLM on the production traits were generally higher than between AMIR and the mean.

**Table 4:** Estimated genetic correlations (SE)

	${r}_{ABIR,m}$	${r}_{ABIR,d}$	${r}_{CMIR,m}$	${r}_{CMIR,d}$	${r}_{m,d}$
WW	− 0.35 (0.11)	− 0.12 (0.16)	− 0.26 (0.12)	− 0.15 (0.17)	0.18 (0.09)
RIB	0.11 (0.12)	0.14 (0.16)	0.15 (0.13)	0.09 (0.17)	0.87 (0.03)
RUMP	0.06 (0.12)	0.00 (0.15)	0.16 (0.13)	0.12 (0.16)	0.90 (0.03)
EMA	− 0.13 (0.12)	− 0.34 (0.25)	0.04 (0.13)	− 0.17 (0.26)	0.30 (0.12)

WW, weaning weight; RIB, rib fat; RUMP, rump fat; EMA, eye muscle area; AMIR, antibody-mediated immune response; CMIR, cell-mediated immune response. ${r}_{ABIR,m}$ = genetic correlation between the mean of the DHGLM and AMIR. ${r}_{ABIR,d}$ = genetic correlation between the dispersion of the DHGLM and AMIR. ${r}_{CMIR,m}$ = genetic correlation between the mean of the DHGLM and CMIR. ${r}_{CMIR,d}$ = genetic correlation between the dispersion of the DHGLM and CMIR. ${r}_{m,d}$= genetic correlation between the mean and dispersion of the DHGLM averaged across the models with AMIR or CMIR as IC trait

None of the genetic correlations between AMIR or CMIR and the dispersion of the DHGLM on the production traits were significant. However, the estimates indicated that both AMIR and CMIR were weakly negatively correlated to the dispersion of WW and EMA and weakly positively correlated to RIB. Furthermore, RUMP was weakly positively correlated to CMIR, but not correlated to AMIR.

For WW, the genetic correlation between mean and dispersion was weakly positive. The genetic correlation between the mean and dispersion of EMA was moderately positive. Meanwhile both RIB and RUMP had strongly positive genetic correlations between mean and dispersion of the DHGLM. The genetic correlations between mean and dispersion were consistent when AMIR and CMIR were fitted with the DHGLM trait.

## Discussion

This study estimated genetic variance of immune competence, growth and scan traits, as well as the micro-GES of growth and scan traits, in Australian Angus cattle. The genetic relationship between immune competence and both mean and micro-GES of growth and scan traits were also investigated.

### Immune response, weight, fat, and muscle

The IC dataset analysed in the current study was a more recent version of that analysed in previous studies [1, 3–5, 25]. As expected the heritabilities of AMIR and CMIR reported here were in line with the heritabilities of 0.30–0.42 for AMIR and 0.25–0.33 for CMIR reported in earlier studies of the IC traits [1, 3–5, 25].

The heritabilities of RIB, RUMP and EMA were comparable to the heritabilities previously reported for the traits in Australian beef cattle [26, 27]. However, the heritability of WW found in the current study was considerably higher than the 0.13–0.35 previously reported for Australian beef cattle [26–29]. This was likely due to fitting a DHGLM on WW. Fitting WW using an animal model resulted in similar additive genetic, maternal genetic and permanent environmental variances as found using the DHGLM. However, the residual variance from the animal model was approximately five times larger than the residual variance of the mean part of the DHGLM. For RIB, RUMP and EMA the residual variance of the animal models was only 1.5–3 times larger than the residual variance of the mean part of the DHGLM. This shows that fitting the DHGLM had a larger impact on the heritability of the mean for WW than for the other production traits.

All the production traits in the current study had genetic variation and heritabilities of the dispersion differing significantly from zero. This study is the first to estimate micro-GES in WW, RIB, RUMP, and EMA in Australian beef cattle. However, significant micro-GES have previously been reported for yearling weight in Australian Angus [30]. The heritability of WW on the logarithmic scale was within the range of those reported for yearling weight, while on the measurement scale the heritability of WW was lower [30]. This could be due to the inclusion of maternal effects for WW in the current study but not for yearling weight in the study by Madsen et al. [30].

The micro-GES of the IC traits were not estimated in the current study, due to the limited number of offspring per sire. Accurate estimation of variance components from DHGLMs require a data structure with a minimum of 500 sires when the average number of offspring per sire is 20 [31]. The current IC dataset falls short on both of these parameters, with only 264 sires and an average number of offspring per sire of 15. Once sufficient IC records are collected, micro-GES of the IC traits should be estimated.

### Resource allocation between immune response and weight, fat, or muscle

The results show some support for the theory that animals with higher IC divert resources away from growth. The genetic correlations between WW and either IC trait were negative, i.e., there was a tendency for animals with higher WW to have lower IC. This is in line with the genetic correlations of − 0.24–0.38 (SE 0.13–0.15) between WW and IC traits or IC indexes previously reported [5]. Assuming that resources are limited, the negative genetic correlation may stem from high IC animals allocating more resources for IC than low IC animals, thus having less resources available for growth [32].

The genetic correlations between EMA and either AMIR or CMIR were low, non-significant and differed in sign. However, it should be noted that the bivariate animal model for EMA with either IC trait estimated significant moderately negative genetic correlations between EMA and both AMIR and CMIR. Reverter et al. [5] likewise found weakly to moderately negative genetic correlations between carcass eye muscle area and IC traits or IC indexes when fitting a bivariate animal model. A negative genetic correlation between EMA and IC traits makes biological sense when considering that EMA is a measure of muscle size, i.e., influenced by growth. As for WW, IC may direct resources away from EMA causing the negative genetic correlation.

In contrast, the results did not indicate any resource competition between the IC and fat traits. The genetic correlations between RIB or RUMP with either AMIR or CMIR were weakly positive. Reverter et al. [5] likewise found carcass rib fat depth to be weakly genetic correlated (− 0.04–0.23, SEs 0.15–0.16) to IC traits or indexes. The low non-significant genetic correlations between the IC traits and RIB and RUMP were somewhat surprising, as multiple studies have shown connections between body fat and immunity. Both low and excessive body fat can impair immune function [33–36]. With this in mind, one might expect a stronger genetic relationship between RIB or RUMP and IC in cattle. However, the genetic potential for subcutaneous fat in cattle may not be related to their immune function unless animals are either extremely lean or obese. It is also possible that characteristics of immune system function that influence body fat composition are not assessed as part of IC testing in Australian Angus.

### Selection on weight, fat, muscle and immune competence traits

Currently, breeding goals generally aim to increase WW and EMA, while aiming to decrease RIB and RUMP. IC traits are not directly included in most breeding schemes yet. The inclusion of IC traits into the selection process requires understanding the relationships between IC traits and the traits currently selected on.

The weak correlations between the IC traits and RIB or RUMP, suggest that selection on RIB and RUMP would have minimal impact on the genetic potential for IC in the population and vice versa. Furthermore, simultaneous selection on RIB or RUMP and either AMIR or CMIR would be possible regardless of the selection direction of the various traits.

In contrast, the negative genetic correlations observed between WW and the IC traits are unfavourable for breeding programs. Increasing WW without accounting for the correlation would decrease IC, which could result in animals that are more prone to serious illness. However, the genetic correlation is moderate, thus identifying animals with high WW and high IC for use in breeding programs is possible. Similarly, a moderately negative genetic correlation between IC and EMA requires identification of animals that can improve both traits in breeding programs.

Another important consideration when including IC traits into a selection index is the type of selection. There is no doubt that low IC animals are undesirable, regardless of their performance for other traits, as these animals may require therapeutic intervention to survive. The low IC animals can also act as disease reservoirs wherein diseases can be maintained indefinitely increasing the incidence of diseases [37]. This might favour a directional selection approach towards higher IC animals. However, high immune responses are energy consuming and may have adverse impacts on other traits. Hine et al. [1] showed that phenotypically high AMIR animals had significantly lower weight gain over the weaning period than animals with average or low phenotypic AMIR. Thus applying directional selection to increase AMIR may not be the optimal strategy.

An alternative approach to increase the IC of the population may be to define a threshold value for the IC traits and remove animals falling below this value [1]. Hine et al. [25] showed that low IC steers had higher mortality rates than average or high IC steers and the health-related costs of low IC steers was more than four times larger than health-related costs of the average and high IC steers. Results like those show that removing the low IC animals could increase profits considerably.

Lastly, the type of IC trait to include in the selection also needs careful consideration. Overall IC of animals is better described by including both measures of AMIR and CMIR in an index as AMIR and CMIR measures two different aspects of IC. Furthermore, their genetic correlation has been estimated in the range of 0.14–0.40 (SE 0.03–0.22) [1, 3–5]. For these reasons, AMIR and CMIR have been combined into an IC index, termed ImmuneDEX [5]. The ImmuneDEX have been shown to have consistently strong genetic correlations (0.68–0.85, SEs 0.04–0.05) to both AMIR and CMIR and can be used for optimal balanced selection on animals to enhance the IC of the population [4, 5]. The ImmuneDEX was not calculated here, as the aim of this study was to estimate the relationship between the individual components of ImmuneDEX and the micro-GES of production traits.

### Selection on micro-genetic environmental sensitivity of weight, fat, and muscle

There are multiple reasons for wanting to reduce the variability of weight, fat, and muscle traits in beef production. Animals with less micro-GES are expected to be less sensitive to minor disturbances [38] and have more uniform performance and end-products [19], which are both desirable for any trait and population. Furthermore, micro-GES of traits with intermediate optimums have been found to have direct economic value [39], as high micro-GES can result in many animals deviating substantially from the optimum even if the population mean is at the optimum.

For WW and EMA, the primary reason for reducing micro-GES would be ensuring product uniformity. Both WW and EMA are under directional selection for increased weight and cut size. For WW the direction selection is due to its downstream impacts on the carcass weight of the animals. Micro-GES of carcase weight itself has not yet been reported, but reducing the variability of WW, and other live weight traits, could help reduce the micro-GES of carcass weight.

For RIB and RUMP, consumers generally favour intramuscular fat over subcutaneous fat and selection is consequently aiming for reduction. However, there is a biological lower limit beyond which reducing body fat, including subcutaneous fat, may negatively impact health or fertility. In dairy cattle, body condition score is used as an indicator of body fat reserves and low body condition scores are unfavourably correlated to fertility [40]. RIB and RUMP may therefore shift from being under directional selection to stabilising selection as the means approach this lower limit of functionality.

The genetic coefficient of variation of the dispersion was considerable compared to the genetic coefficient of variation of the mean for all traits. This, along with the significant heritabilities of the dispersion, showed that selection on micro-GES of WW, RIB, RUMP, and EMA should be possible. Divergent selection lines have been successfully established for variability in birth weight in mice from a base population with a lower heritability and genetic coefficient of variation of the dispersion than found for any trait in the current study [41]. In the study by Formoso‐Rafferty et al. [41] selection responses over 7 generations were approximately 0.35 ln^2^g in the high line and − 0.30 ln^2^g in the low line. It should therefore be possible to alter the micro-GES of WW, RIB, RUMP, and EMA through selection. However, it should be noted that the heritabilities of the dispersion on the measurement scale were low for all traits, and accuracies of EBVs, and consequently selection, would be low.

When considering whether to select on micro-GES of a trait, it is important to assess the potential impacts of selection on micro-GES on other traits. The genetic correlations between mean and dispersion of the DHGLM on WW indicate that animals with high WW tend to also have more micro-GES than animals with low WW. The same pattern was observed for EMA. Selection for increased performance in WW or EMA would therefore be expected to increase the micro-GES of WW or EMA in the population. The genetic correlations between WW and micro-GES of WW or EMA and micro-GES of EMA are however low so the correlated response would be small relative to the direct response in WW or EMA.

A reduction in micro-GES of RIB or RUMP would result in a correlated reduction in mean performance of RIB or RUMP, due to the strongly positive genetic correlation between mean and dispersion for both RIB and RUMP. Seen from a consumer standpoint this correlation is favourable, but as mentioned, there may be a biological limit on leanness. If the selection to reduce micro-GES pushes RIB and RUMP below this limit, it may result in adverse impacts on the fertility and health of the animals. This should be carefully considered when selecting to reduce micro-GES of RIB and RUMP.

In most breeding programs animals are selected based on an index rather than their EBVs for a single trait. It has been shown that heritabilities of at least 0.02 are required when using index selection for simultaneous improvement of mean (index weight of 1) and dispersion (index weight of − 1) of a trait in a sib testing scheme [39]. A selection index based on both the mean and dispersion of WW, RIB, RUMP, or EMA could therefore be successful. However, more research is needed into the requirements when including micro-GES of traits in selection indexes with many other traits and possibly with micro-GES on more than one trait.

### Immune competence and micro-genetic environmental sensitivity

This study is the first to report genetic correlations between IC traits and micro-GES. The genetic correlations were not significantly different from zero for any trait combination as the SEs were relatively large for all estimates. SEs depend on data size, and it is possible that with more IC records the genetic correlations may become significant. The estimated genetic correlations presented in the current study are thus preliminary and can only inform about the expected trends.

The estimated genetic correlations between micro-GES of WW or EMA and IC were weakly negative, thus some animals with low micro-GES in WW or EMA tended to have higher IC. These correlations are favourable to breeding programs, as selecting for reduced micro-GES in WW or EMA could improve IC. Meanwhile, micro-GES of RIB and RUMP tended to be weakly positively correlated with IC, meaning some animals with high IC tended to have high micro-GES of RIB and RUMP. In this case reducing micro-GES of RIB and RUMP might lead to a reduction in IC, which is unfavourable. Clearly, the relationship between IC traits and micro-GES depends on the trait exhibiting micro-GES.

To some extent, the low-moderate genetic correlations between micro-GES and IC traits found in the current study were not surprising. While it could be expected that animals with low IC would have higher micro-GES, it is possible that animals differ in which traits an immune challenge would cause variation in. If this is the case, a sire family with low IC may have more variable phenotypes for WW, but not for RIB, while the opposite holds for a different sire family. This would lead to low or moderate genetic correlations between micro-GES and IC. Micro-GES and IC thus represent two different forms of sensitivity in a population and selection to improve both should be considered in breeding programs as means of improving health, welfare, and product uniformity in livestock production.

## Conclusions

This study investigated the variance of micro-GES in WW, RIB, RUMP, and EMA as well as the genetic relationship between AMIR or CMIR and both mean and micro-GES of WW, RIB, RUMP and EMA in Australian Angus cattle.

Micro-GES was found in WW, RIB, RUMP and EMA and the dispersion was found to have considerable genetic coefficients of variation compared to the genetic coefficients of variation of WW, RIB, RUMP, and EMA. Selection to increase uniformity and decrease general sensitivity may be possible for WW, RIB, RUMP, and EMA. However, the heritability of the dispersion was low, and the accuracy of selection can therefore be expected to be low.

Significant unfavourable genetic correlations were found between the IC traits and WW. This supported the hypothesis that mounting ICs directs resources away from growth and vice versa. The genetic correlations between IC traits and the production traits or their micro-GES were associated with large SEs, such that only the genetic correlations between the IC traits and WW were significant. The large SEs were attributed to the small dataset of AMIR and CMIR and would be expected to decrease with increasing dataset size. Based on the non-significant genetic correlations between the IC traits and micro-GES of production traits it seems likely that micro-GES and IC measures two different forms of sensitivity. Selection to improve both micro-GES and IC should be considered in breeding programs as means of improving health, welfare, and product uniformity in livestock production.

## Supplementary Information

**Additional file 1.** How to run a DHGLM using R and DMU. A guide on the general method for implementing the algorithm given in the Statistical analysis section of the Methods using R and DMU.

**Additional file 2: Table S1.** Estimates obtained from the bivariate animal models. **Table S2.** Estimates obtained from the bivariate sire models. **Table S3.** Estimates obtained from the trivariate models with a double hierarchical generalised linear model prior to correction for the use of sire models.

## Data Availability

Any data request should be made directly to CSIRO for immune response data and Angus Australia for production data.
