# Supplementary material for: The genetic relationship between immune competence traits and micro-genetic environmental sensitivity of weight, fat, and muscle traits in Australian Angus cattle
Source: Genet Sel Evol. 2025 Sep 25;57:47. doi: 10.1186/s12711-025-00998-8 (PMC12465796; doi:10.1186/s12711-025-00998-8)
Supplement: Supplementary file 1 — Additional file 1. How to run a DHGLM using R and DMU. A guide on the general method for implementing the algorithm given in the Statistical analysis section of the Methods using R and DMU. [file 12711_2025_998_MOESM1_ESM.docx]

# Additional file 1 – How to run a DHGLM using R and DMU.

It is assumed the reader is familiar with general data editing and calculations in R and analysing data using the DMU software.

The scripts have been annotated with comments to improve understanding. It is necessary to remove comments (#) from the lines of the DMU scripts that start with ‘$’, otherwise the request will not be carried out correctly.

## Abbreviations

PT = PT for which mean and dispersion variances are to be estimated.

IC = IC for which mean variances are to be estimated.

yd = dispersion phenotype for PT

Wd = weight for the residual of the dispersion for PT

W = weight for the residual of the mean for PT

## R-script

##Preperations##

setwd('PathToAnalysis') #replace PathToAnalysis with relevant information

#Load packages

require('data.table') #Install if not already available. Alternatively read.table() can replace fread()

#Read phenotype file

dat<-fread('PathToFile/data.txt')

#Replace PathToFile and file name+type with own information

#Perform required data edits such as:

#recoding alphanumeric values to numeric values ect.

#ensuring columns with factors (integers) come before columns with covariates (reals)

#Save cleaned data

fwrite(dat,'data.txt',na='-9999',col.names=F,quote=F)

#Iteration setup - defining the parameters for the algorithm to run the DHGLM

i <- 1 #Starting value for the iteration counter

eps <- 1e-4 # Convergence limit

delta <- 1 #Initial delta value

niter <- 50 # Max iterations of the algorithm

n <- 3 #Number of traits including the dispersion (here IC and PT trait and dispersion of PT trait)

results <- matrix(ncol=n*(n+1)/2, nrow=niter) # allocate space for variance-covariance outputs from analysis

##ALGORITHM##

**#Step 1: Run linear mixed model on PT and IC**

#Example dir file provided after script

**#Step 2: Calculate yd (Eq.5) and Wd (Eq.2)**

res <- read.table('LMM.RESIDUAL', header=FALSE, na='-9999') #Load residuals from step 1

# V1= record no;

# V2-3=observation for IC and PT trait;

# V4-5=predicted value of IC and PT trait;

# V6-7=residual of IC and PT trait;

# V8-9=leverage of IC and PT trait

res$yd <- (res$V6^2)/(1 - res$V8) #Eq.5

res$Wd <- (1 - res$V8)/2 #Eq.2

dat2 <- cbind(dat, res[match(rownames(dat),res[,1]),c('yd','Wd')]) #Add yd and wd to data

fwrite(dat2, file='data.txt', na = '-9999', col.names=F) #Save data

**#Step 3: Run a univariate generalized linear mixed model on yd with a log-link function**

#Example dir file provided after script

**#Step 4: Calculate W (Eq.1)**

res <- read.table('GLM.RESIDUAL', header=FALSE, na='-9999')

# V1= record no;

# V2=observation for PT trait;

# V3&4=predicted of PT trait;

# V5=residual of PT trait;

# V6=leverage of PT trait

res$W <- 1/res$V4 #Eq.1

dat2$W<-res$W[match(rownames(dat2),res[,1])] #Add W to data

fwrite(dat2, file='data.txt', na = '-9999', col.names = FALSE) #Save data

**#Step 5-7: Run the DHGLM (step 5), recalculate yd, Wd and W (Step 6) and repeat steps 5 and 6 until convergence or niter reached (Step 7)**

while(delta > eps){ #Set up loop for Step 7 - end if delta less than convergence criteria

if( i > niter) break; #End loop if more than 50 iterations have been completed

#Run DHGLM (Step 5) – We recommend using a system() to call a BASH file (not provided) that runs DMU so this loop can run fully from R.

#Recalculate values (Step 6)

res <- read.table('DHGLM.RESIDUAL', header=FALSE, na='-9999')

# V1= record no;

# V2-4=observation for IC, mean PT and PT dispersion;

# V5-7=linear predicted value of IC, mean PT and PT dispersion;

# V8-10=predicted value of IC, mean PT and PT dispersion;

# V11-13=residual of IC, mean PT and PT dispersion;

# V14-16=deviance residual of IC, mean PT and PT dispersion;

# V17-19=Pearson residual of IC, mean PT and PT dispersion;

# V20-22=leverage of IC, mean PT and PT dispersion

res$yd <- (res$V11^2)/(1 - res$V20) #Eq.5

res$Wd <- (1 - res$V20)/2 #Eq.2

res$W <- 1/res$V9 #Eq.1

dat2$yd <- res$yd[match(rownames(dat2),res[,1])] #Update existing yd values

dat2$Wd <- res$Wd[match(rownames(dat2),res[,1])] #Update existing Wd values

dat2$W <- res$W[match(rownames(dat2),res[,1])] #Update existing W values

fwrite(dat2, file='data.txt', na = '-9999', col.names = FALSE) #Save data

#Store (co)varainces and re-calculate delta

cvov <- read.table('DHGLM.PAROUT', header=FALSE )[1:(n*(n+1)/2), 4] #Load estimated variances

results[i, ] <- cvov #Store estimated variances

delta <- ifelse(i==1, 1, max(abs((results[i - 1,] - results[i, ])/results[i, ])))

print(paste('iteration:', i)); print(paste('Delta:', delta)); print(results[i, ])

i <- i + 1 #increase i

}

## Example DIR file for Step 1 (LMM.dir)

$COMMENT

Example DIR file for linear mixed model step - Sire model

$ANALYSE 1 1 0 0

$DATA ASCII (5,5,-9999) ../data.txt

$VARIABLE

#1 2 3 4 5

Sire Dam Sex CG_PT CG_IC

#1 2 3 4 5

PT IC Age_PT DamAge DamAge2

$MODEL

2 2 0 0 0

0

0

# Model:

1 0 3 3 4 1 #PT=Sex+ContemporaryGroup+Sire (Add other fixed and random effects as required)

2 0 3 3 5 1 #IC=Sex+ContemporaryGroup+Sire (Add other fixed and random effects as required)

# random effect:

1 1 #each trait has one random effect (the last in the model)

1 1 #they are assigned to the same matrix (matrix 1) to obtain the covariance as well as variances

# Regression:

1 3 #Age fitted as covariate in the model for the PT (add others if needed)

0 #no covariate for the IC (add others if needed)

#Missing residual covariances

1 #the traits were not measured on the same animals, the residual covariance is therefor missing

2 1

$VAR_STR 1 PED 6 RANDOM 1.0 ASCII PathToPedigree/ped_sire.txt #Replace PathToPedigree/ped_sire.txt with the relevant information

$RESIDUALS ASCII

#Print the residuals from the model - other parameters (e.g. SOLUTIONS) can be requested as well but are not required

## Example DIR file for Step 3 (GLM.dir)

$COMMENT

Example DIR file for GLM step - Sire model

$ANALYSE 1 1 0 0

$DATA ASCII (5,7,-9999) ../data.txt

$VARIABLE

#1 2 3 4 5

Sire Dam Sex CG_PT CG_IC

#1 2 3 4 5 6 7

PT IC Age_PT DamAge DamAge2 yd wd

$MODEL

1

0

# Model:

6 7 3 3 4 1 #Dispersion of PT=Sex+ContemporaryGroup+Sire (Add other fixed and random effects as required)

# random effect:

1 1 #one random effect (the last in the model)

# Regression:

1 3 #Age fitted as covariate (add others if needed)

#Missing residual covariances

0

$VAR_STR 1 PED 6 RANDOM 1.0 ASCII PathToPedigree/ped_sire.txt #Replace PathToPedigree/ped_sire.txt with the relevant information

$RESIDUALS ASCII

#Print the residuals from the model - other outputs (e.g. SOLUTIONS) can be requested as well but are not required

$GLMM 1 VARF=GAMMA LINK=LOG #Fit a GLMM with a log link function

## Example DIR file for Step 5 (DHGLM.dir)

$COMMENT

Example DIR file for DHGLMM step - Sire model

$ANALYSE 1 1 0 0

$DATA ASCII (5,8,-9999) ../data.txt

$VARIABLE

#1 2 3 4 5

Sire Dam Sex CG_PT CG_IC

#1 2 3 4 5 6 7 8

PT IC Age_PT DamAge DamAge2 yd wd W

$MODEL

3 3 0 0

0

0

0

# Model:

1 8 3 3 4 1 #PT=Sex+ContemporaryGroup+Sire (Add other fixed and random effects as required)

6 7 3 3 4 1 #Dispersion of PT=Sex+ContemporaryGroup+Sire (Add other fixed and random effects as required)

2 0 3 3 5 1 #IC=Sex+ContemporaryGroup+Sire (Add other fixed and random effects as required)

# random effect:

1 1 #each trait has one random effect (the last in the model)

1 1 #they are assigned to the same matrix (matrix 1) to obtain the covariance as well as variances

1 1

# Regression:

1 3 #Age fitted as covariate (add others if needed)

1 3 #Age fitted as covariate (add others if needed)

0 #no covariate for the IC (add others if needed)

#Missing residual covariances

3

2 1

3 1

3 2

$VAR_STR 1 PED 6 RANDOM 1.0 ASCII PathToPedigree/ped_sire.txt #Replace PathToPedigree/ped_sire.txt with the relevant information

$RESIDUALS ASCII

#Print the residuals from the model - other parameters (e.g. SOLUTIONS) can be requested as well but are not required

$GLMM 2 VARF=GAMMA LINK=LOG #Fit a GLMM with a log link function for the dispersion of the PT
