# Supplementary material for: The genetic relationship between immune competence traits and micro-genetic environmental sensitivity of weight, fat, and muscle traits in Australian Angus cattle
Source: Genet Sel Evol. 2025 Sep 25;57:47. doi: 10.1186/s12711-025-00998-8 (PMC12465796; doi:10.1186/s12711-025-00998-8)
Supplement: Supplementary file 2 — Additional file 2: Table S1. Estimates obtained from the bivariate animal models. Table S2. Estimates obtained from the bivariate sire models. Table S3. Estimates obtained from the trivariate models with a double hierarchical generalised linear model prior to correction for the use of sire models. [file 12711_2025_998_MOESM2_ESM.docx]

## Additional file 2 – Supplementary Tables

Table S1: Estimates from the bivariate animal models.

|  | **WW** | **RIB** | **P8** | **EMA** |
| --- | --- | --- | --- | --- |
| **Models fitted with AMIR as IC trait** | | | | |
| $\boldsymbol{\sigma}_{\boldsymbol{a}_{\boldsymbol{m}}}^{\mathbf{2}}$ | 122.5184 (9.8731) | 0.6323 (0.0239) | 1.5333 (0.0526) | 8.3785 (0.3778) |
| $\boldsymbol{\sigma}_{\boldsymbol{a}_{\boldsymbol{AMIC}}}^{\mathbf{2}}$ | 0.0180 (0.0025) | 0.0184 (0.0025) | 0.0184 (0.0025) | 0.0188 (0.0025) |
| $\boldsymbol{\sigma}_{\boldsymbol{c}}^{\mathbf{2}}$ | 40.9905 (6.158) |  |  |  |
| $\boldsymbol{\sigma}_{\boldsymbol{pe}}^{\mathbf{2}}$ | 77.4818 (6.6681) |  |  |  |
| $\boldsymbol{\sigma}_{\boldsymbol{e}_{\boldsymbol{m}}}^{\mathbf{2}}$ | 289.0866 (7.337) | 1.5860 (0.0184) | 3.1022 (0.0391) | 28.3945 (0.3031) |
| $\boldsymbol{\sigma}_{\boldsymbol{e}_{\boldsymbol{AMIR}}}^{\mathbf{2}}$ | 0.0279 (0.0020) | 0.0275 (0.0021) | 0.0275 (0.0021) | 0.0273 (0.0021) |
| $\boldsymbol{\sigma}_{\boldsymbol{a}_{\boldsymbol{m}}\boldsymbol{,}\boldsymbol{a}_{\boldsymbol{AMIR}}}$ | -0.3746 (0.089) | 0.0041 (0.0077) | 0.0106 (0.0109) | -0.0927 (0.0324) |
| $\boldsymbol{r}_{\boldsymbol{a}_{\boldsymbol{m}}\boldsymbol{,}\boldsymbol{a}_{\boldsymbol{AMIR}}}$ | -0.2523 (0.0611) | 0.0383 (0.0715) | 0.0628 (0.0649) | -0.2339 (0.0809) |
| **Models fitted with CMIR as IC trait** | | | | |
| $\boldsymbol{\sigma}_{\boldsymbol{a}_{\boldsymbol{m}}}^{\mathbf{2}}$ | 183.6108 (17.98) | 0.6623 (0.0243) | 1.6009 (0.0534) | 9.2257 (0.3917) |
| $\boldsymbol{\sigma}_{\boldsymbol{a}_{\boldsymbol{CMIC}}}^{\mathbf{2}}$ | 0.0183 (0.0040) | 0.0227 (0.0056) | 0.0244 (0.0054) | 0.0245 (0.0053) |
| $\boldsymbol{\sigma}_{\boldsymbol{c}}^{\mathbf{2}}$ | 39.9430 (9.908) |  |  |  |
| $\boldsymbol{\sigma}_{\boldsymbol{pe}}^{\mathbf{2}}$ | 86.3650 (13.0883) |  |  |  |
| $\boldsymbol{\sigma}_{\boldsymbol{e}_{\boldsymbol{m}}}^{\mathbf{2}}$ | 291.1263 (14.54) | 1.5803 (0.0185) | 3.0978 (0.0395) | 28.2724 (0.3098) |
| $\boldsymbol{\sigma}_{\boldsymbol{e}_{\boldsymbol{CMIR}}}^{\mathbf{2}}$ | 0.0280 (0.0035) | 0.0254 (0.0047) | 0.0240 (0.0045) | 0.0239 (0.0044) |
| $\boldsymbol{\sigma}_{\boldsymbol{a}_{\boldsymbol{m}}\boldsymbol{,}\boldsymbol{a}_{\boldsymbol{CMIR}}}$ | -0.4942 (0.140) | 0.0059 (0.0091) | 0.0122 (0.0126) | -0.1071 (0.0397) |
| $\boldsymbol{r}_{\boldsymbol{a}_{\boldsymbol{m}}\boldsymbol{,}\boldsymbol{a}_{\boldsymbol{CMIR}}}$ | -0.2696 (0.0805) | 0.0480 (0.0747) | 0.0616 (0.0639) | -0.2251 (0.0840) |

WW = weaning weight. RIB = rib fat. RUMP = rump fat. EMA = eye muscle area. AMIR = antibody-mediated immune response. CMIR = cell-mediated immune response. $\sigma_{a_{m}}^{2}$ and$\sigma_{e_{m}}^{2}$ = additive genetic variance and residual variance of WW, RIB, RUMP, and EMA. $\sigma_{a_{AMIR}}^{2}$ and$\sigma_{e_{AMIR}}^{2}$ = additive genetic variance and residual variance of AMIR. $\sigma_{a_{CMIR}}^{2}$ and$\sigma_{e_{CMIR}}^{2}$ = additive genetic variance and residual variance of CMIR. $\sigma_{a_{m},a_{AMIR}}$ and $\sigma_{a_{m},a_{CMIR}}$ = covariance between the additive genetic variance of WW, RIB, RUMP or EMA and AMIR or CMIR. $\sigma_{c}^{2}$and $\sigma_{pe}^{2}$ = the maternal genetic and permanent environmental variance, respectively, of WW.

Table S2: Estimates from the bivariate sire models.

|  | **WW** | **RIB** | **P8** | **EMA** |
| --- | --- | --- | --- | --- |
| **Models fitted with AMIR as IC trait** | | | | |
| $\boldsymbol{\sigma}_{\boldsymbol{s}_{\boldsymbol{m}}}^{\mathbf{2}}$ | 38.5243 (3.3267) | 0.1612 (0.0096) | 0.3514 (0.0204) | 2.1040 (0.1377) |
| $\boldsymbol{\sigma}_{\boldsymbol{s}_{\boldsymbol{AMIC}}}^{\mathbf{2}}$ | 0.0041 (0.0007) | 0.0041 (0.0007) | 0.0041 (0.0007) | 0.0042 (0.0007) |
| $\boldsymbol{\sigma}_{\boldsymbol{c}}^{\mathbf{2}}$ | 61.4345 (6.4749) |  |  |  |
| $\boldsymbol{\sigma}_{\boldsymbol{pe}}^{\mathbf{2}}$ | 82.1866 (7.0065) |  |  |  |
| $\boldsymbol{\sigma}_{\boldsymbol{e}_{\boldsymbol{m}}}^{\mathbf{2}}$ | 349.7539 (5.1022) | 2.0395 (0.0103) | 4.2013 (0.0212) | 34.3442 (0.1732) |
| $\boldsymbol{\sigma}_{\boldsymbol{e}_{\boldsymbol{AMIR}}}^{\mathbf{2}}$ | 0.0416 (0.0010) | 0.0416 (0.0010) | 0.0416 (0.0010) | 0.0416 (0.0010) |
| $\boldsymbol{\sigma}_{\boldsymbol{s}_{\boldsymbol{m}}\boldsymbol{,}\boldsymbol{s}_{\boldsymbol{AMIR}}}$ | -0.1352 (0.0478) | 0.0023 (0.0032) | 0.0017 (0.0047) | -0.0193(0.0123) |
| $\boldsymbol{r}_{\boldsymbol{s}_{\boldsymbol{m}}\boldsymbol{,}\boldsymbol{s}_{\boldsymbol{AMIR}}}$ | -0.3385 (0.1139) | 0.0908 (0.1229) | 0.0440 (0.1229) | -0.2058 (0.1288) |
| **Models fitted with CMIR as IC trait** | | | | |
| $\boldsymbol{\sigma}_{\boldsymbol{s}_{\boldsymbol{m}}}^{\mathbf{2}}$ | 38.7559 (3.3380) | 0.1612 (0.0096) | 0.3515 (0.0204) | 2.1088 (0.1379) |
| $\boldsymbol{\sigma}_{\boldsymbol{s}_{\boldsymbol{CMIC}}}^{\mathbf{2}}$ | 0.0031 (0.0005) | 0.0032 (0.0005) | 0.0032 (0.0005) | 0.0032 (0.0005) |
| $\boldsymbol{\sigma}_{\boldsymbol{c}}^{\mathbf{2}}$ | 61.3888 (6.4726) |  |  |  |
| $\boldsymbol{\sigma}_{\boldsymbol{pe}}^{\mathbf{2}}$ | 82.3501 (7.0059) |  |  |  |
| $\boldsymbol{\sigma}_{\boldsymbol{e}_{\boldsymbol{m}}}^{\mathbf{2}}$ | 349.6017 (5.1006) | 2.0395 (0.0103) | 4.2013 (0.0212) | 34.3432 (0.1731) |
| $\boldsymbol{\sigma}_{\boldsymbol{e}_{\boldsymbol{CMIR}}}^{\mathbf{2}}$ | 0.0322 (0.0008) | 0.0322 (0.0008) | 0.0322 (0.0008) | 0.0322 (0.0008) |
| $\boldsymbol{\sigma}_{\boldsymbol{s}_{\boldsymbol{m}}\boldsymbol{,}\boldsymbol{s}_{\boldsymbol{CMIR}}}$ | -0.0847 (0.0423) | 0.0040 (0.0029) | 0.0046 (0.0042) | 0.0003 (0.0112) |
| $\boldsymbol{r}_{\boldsymbol{s}_{\boldsymbol{m}}\boldsymbol{,}\boldsymbol{s}_{\boldsymbol{CMIR}}}$ | -0.2427 (0.1185) | 0.1755 (0.1250) | 0.1381 (0.1254) | 0.0043 (0.1367) |

WW = weaning weight. RIB = rib fat. RUMP = rump fat. EMA = eye muscle area. AMIR = antibody-mediated immune response. CMIR = cell-mediated immune response. $\sigma_{s_{m}}^{2}$ and$\sigma_{e_{m}}^{2}$ = additive genetic sire variance and residual variance of WW, RIB, RUMP, and EMA. $\sigma_{s_{AMIR}}^{2}$ and$\sigma_{e_{AMIR}}^{2}$ = additive genetic sire variance and residual variance of AMIR. $\sigma_{s_{CMIR}}^{2}$ and$\sigma_{e_{CMIR}}^{2}$ = additive genetic sire variance and residual variance of CMIR. $\sigma_{s_{m},s_{AMIR}}$ and $\sigma_{s_{m},s_{CMIR}}$ = covariance between the additive genetic sire variance of WW, RIB, RUMP or EMA and AMIR or CMIR. $\sigma_{c}^{2}$and $\sigma_{pe}^{2}$ = the maternal genetic and permanent environmental variance, respectively, of WW.

Table S3: Raw estimates from trivariate models with a double hierarchical generalised linear model.

|  | **WW** | **RIB** | **P8** | **EMA** |
| --- | --- | --- | --- | --- |
| **Models fitted with CMIR as IC trait** | | | | |
| $\boldsymbol{\sigma}_{\boldsymbol{s}_{\boldsymbol{m}}}^{\mathbf{2}}$ | 31.8950 (2.7197) | 0.0805 (0.0049) | 0.1756 (0.0102) | 1.5849 (0.1001) |
| $\boldsymbol{\sigma}_{\boldsymbol{s}_{\boldsymbol{d}}}^{\mathbf{2}}$ | 0.0373 (0.0068) | 0.0268 (0.0031) | 0.0324 (0.0034) | 0.0050 (0.0016) |
| $\boldsymbol{\sigma}_{\boldsymbol{s}_{\boldsymbol{CMIC}}}^{\mathbf{2}}$ | 0.0041 (0.0007) | 0.0042 (0.0007) | 0.0041 (0.0007) | 0.0041 (0.0007) |
| $\boldsymbol{\sigma}_{\boldsymbol{c}}^{\mathbf{2}}$ | 57.5926 (5.4433) |  |  |  |
| $\boldsymbol{\sigma}_{\boldsymbol{pe}}^{\mathbf{2}}$ | 61.0003 (5.2815) |  |  |  |
| $\boldsymbol{\sigma}_{\boldsymbol{e}_{\boldsymbol{m}}}^{\mathbf{2}}$ | 0.9998 (0.0131) | 0.9999 (0.0050) | 0.9999 (0.0050) | 1.0000 (0.0050) |
| $\boldsymbol{\sigma}_{\boldsymbol{e}_{\boldsymbol{d}}}^{\mathbf{2}}$ | 0.8282 (0.0067) | 0.8955 (0.0045) | 0.9116 (0.0046) | 0.9564 (0.0048) |
| $\boldsymbol{\sigma}_{\boldsymbol{e}_{\boldsymbol{CMIR}}}^{\mathbf{2}}$ | 0.0416 (0.0010) | 0.0416 (0.0010) | 0.0416 (0.010) | 0.0416 (0.0010) |
| $\boldsymbol{\sigma}_{\boldsymbol{s}_{\boldsymbol{m}}\boldsymbol{,}\boldsymbol{s}_{\boldsymbol{d}}}$ | 0.1991 (0.1016) | 0.0404 (0.0031) | 0.0679 (0.0048) | 0.0264 (0.0107) |
| $\boldsymbol{\sigma}_{\boldsymbol{s}_{\boldsymbol{m}}\boldsymbol{,}\boldsymbol{s}_{\boldsymbol{CMIR}}}$ | -0.1275 (0.0434) | 0.0019 (0.0023) | 0.0016 (0.0033) | -0.0107 (0.0099) |
| $\boldsymbol{\sigma}_{\boldsymbol{s}_{\boldsymbol{d}}\boldsymbol{,}\boldsymbol{s}_{\boldsymbol{CMIR}}}$ | -0.0015 (0.0020) | 0.0015 (0.0017) | 0.0000 (0.0018) | -0.0015 (0.0011) |
| $\boldsymbol{r}_{\boldsymbol{s}_{\boldsymbol{m}}\boldsymbol{,}\boldsymbol{s}_{\boldsymbol{d}}}$ | 0.1827 (0.0920) | 0.8703 (0.0330) | 0.9003 (0.0281) | 0.2965 (0.1188) |
| $\boldsymbol{r}_{\boldsymbol{s}_{\boldsymbol{m}}\boldsymbol{,}\boldsymbol{s}_{\boldsymbol{CMIR}}}$ | -0.3515 (0.1136) | 0.1050 (0.1229) | 0.0595 (0.1227) | -0.1322 (0.1220) |
| $\boldsymbol{r}_{\boldsymbol{s}_{\boldsymbol{d}}\boldsymbol{,}\boldsymbol{s}_{\boldsymbol{CMIR}}}$ | -0.1203 (0.1622) | 0.1411 (0.1580) | 0.0016 (0.1545) | -0.3410 (0.2494 |
| $tr\left( \boldsymbol{W} \right)/n$ | 0.0066 | 1.1714 | 0.5481 | 0.0422 |
| $tr\left( \boldsymbol{W}_{\boldsymbol{d}} \right)/n$ | 0.3662 | 0.4797 | 0.4794 | 0.4795 |
| **Models fitted with CMIR as IC trait** | | | | |
| $\boldsymbol{\sigma}_{\boldsymbol{s}_{\boldsymbol{m}}}^{\mathbf{2}}$ | 32.1056 (2.7301) | 0.0806 (0.0049) | 0.1755 (0.0102) | 1.5881 (0.1003) |
| $\boldsymbol{\sigma}_{\boldsymbol{s}_{\boldsymbol{d}}}^{\mathbf{2}}$ | 0.0376 (0.0068) | 0.0265 (0.0031) | 0.0325 (0.0.34) | 0.0050 (0.0016) |
| $\boldsymbol{\sigma}_{\boldsymbol{s}_{\boldsymbol{CMIC}}}^{\mathbf{2}}$ | 0.0031 (0.0005) | 0.0032 (0.0005) | 0.0031 (0.0005) | 0.0032 (0.0005) |
| $\boldsymbol{\sigma}_{\boldsymbol{c}}^{\mathbf{2}}$ | 57.5554 (5.4421) | |  |  |
| $\boldsymbol{\sigma}_{\boldsymbol{pe}}^{\mathbf{2}}$ | 61.0376 (5.2816) | |  |  |
| $\boldsymbol{\sigma}_{\boldsymbol{e}_{\boldsymbol{m}}}^{\mathbf{2}}$ | 0.9998 (0.0131) | 0.9999 (0.0050) | 0.99993 (0.0050) | 1.0000 (0.0050) |
| $\boldsymbol{\sigma}_{\boldsymbol{e}_{\boldsymbol{d}}}^{\mathbf{2}}$ | 0.8285 (0.0067) | 0.8955 (0.0045) | 0.91158 (0.0046) | 0.9565 (0.0048) |
| $\boldsymbol{\sigma}_{\boldsymbol{e}_{\boldsymbol{CMIR}}}^{\mathbf{2}}$ | 0.0322 (0.0008) | 0.0322 (0.0008) | 0.03220 (0.0008) | 0.0322 (0.0008) |
| $\boldsymbol{\sigma}_{\boldsymbol{s}_{\boldsymbol{m}}\boldsymbol{,}\boldsymbol{s}_{\boldsymbol{d}}}$ | 0.2024 (0.1020) | 0.0403 (0.0031) | 0.0680 (0.0048) | 0.0272 (0.0107) |
| $\boldsymbol{\sigma}_{\boldsymbol{s}_{\boldsymbol{m}}\boldsymbol{,}\boldsymbol{s}_{\boldsymbol{CMIR}}}$ | -0.0820 (0.0382) | 0.0024 (0.0020) | 0.0038 (0.0030) | 0.0028 (0.0090) |
| $\boldsymbol{\sigma}_{\boldsymbol{s}_{\boldsymbol{d}}\boldsymbol{,}\boldsymbol{s}_{\boldsymbol{CMIR}}}$ | -0.0016 (0.0018) | 0.0008 (0.0015) | 0.0012 (0.0016) | -0.0007 (0.0010) |
| $\boldsymbol{r}_{\boldsymbol{s}_{\boldsymbol{m}}\boldsymbol{,}\boldsymbol{s}_{\boldsymbol{d}}}$ | 0.1843 (0.0916) | 0.8711 (0.1491) | 0.9002 (0.0280) | 0.3051 (0.1185) |
| $\boldsymbol{r}_{\boldsymbol{s}_{\boldsymbol{m}}\boldsymbol{,}\boldsymbol{s}_{\boldsymbol{CMIR}}}$ | -0.2586 (0.1173) | 0.1491 (0.1272) | 0.1629 (0.1254) | 0.0399 (0.1264) |
| $\boldsymbol{r}_{\boldsymbol{s}_{\boldsymbol{d}}\boldsymbol{,}\boldsymbol{s}_{\boldsymbol{CMIR}}}$ | -0.1518 (0.1660) | 0.0870 (0.1679) | 0.1192 (0.1592) | -0.1666 (0.2632) |
| $tr\left( \boldsymbol{W} \right)/n$ | 0.0067 | 1.1776 | 0.54810 | 0.0423 |
| $tr\left( \boldsymbol{W}_{\boldsymbol{d}} \right)/n$ | 0.3662 | 0.4797 | 0.47936 | 0.4795 |

WW = weaning weight. RIB = rib fat. RUMP = rump fat. EMA = eye muscle area. AMIR = antibody-mediated immune response. CMIR = cell-mediated immune response. $\sigma_{s_{m}}^{2}$ and$\sigma_{e_{m}}^{2}$ = additive genetic sire variance and residual variance of the mean of WW, RIB, RUMP, and EMA. $\sigma_{s_{d}}^{2}$ and$\sigma_{e_{d}}^{2}$ = additive genetic sire variance and residual variance of the dispersion of WW, RIB, RUMP, and EMA. $\sigma_{s_{AMIR}}^{2}$ and$\sigma_{e_{AMIR}}^{2}$ = additive genetic sire variance and residual variance of AMIR. $\sigma_{s_{m},s_{d}}$ = covariance between the additive genetic sire variance of the mean and dispersion of WW, RIB, RUMP, or EMA. $\sigma_{s_{CMIR}}^{2}$ and$\sigma_{e_{CMIR}}^{2}$ = additive genetic sire variance and residual variance of CMIR. $\sigma_{s_{d},s_{AMIR}}$ and $\sigma_{s_{d},s_{CMIR}}$ = covariance between the additive genetic sire variance of the mean of WW, RIB, RUMP or EMA and AMIR or CMIR. $\sigma_{s_{m},s_{AMIR}}$ and $\sigma_{s_{m},s_{CMIR}}$ = covariance between the additive genetic sire variance of the dispersion of WW, RIB, RUMP or EMA and AMIR or CMIR. $\sigma_{c}^{2}$and $\sigma_{pe}^{2}$ = the maternal genetic and permanent environmental variance, respectively, of WW. $tr\left( \boldsymbol{W} \right)/n$ and $tr\left( \boldsymbol{W}_{\boldsymbol{d}} \right)/n$ = the average weightings for the residual variance of the mean and dispersion of WW, RIB, RUMP, and EMA.
